# How did medicaid expansions affect labor supply and welfare enrollment? Evidence from the early 2000s

**DOI:** 10.1186/s13561-016-0089-3

**Published:** 2016-03-22

**Authors:** Cagdas Agirdas

**Affiliations:** Sykes College of Business, University of Tampa, Box O, 401 W. Kennedy Blvd, Tampa, FL 33606 USA

**Keywords:** Medicaid, Labor supply, Food stamps, I11, I13

## Abstract

In the early 2000s, Arizona, Maine, New Mexico, New York, Oregon, and Vermont expanded Medicaid to cover more low-income individuals, primarily childless adults. This change provides the researcher with an opportunity to analyze the effects of these expansions on labor supply and welfare enrollment. I use a large data set of 176 counties over 7 years, including 3 years of pre-expansion period, 1 year of implementation year, and 3 years of post-expansion period. Using a difference-in-differences approach, I find the most-affected counties had a 1.4 percentage-point more decline in labor force participation rate in comparison to other counties. Furthermore, I observe a 0.32 h decrease in average weekly hours and a 1.1 % increase in average weekly wages. This indicates labor supply was affected more than labor demand. I also observe a 0.49 % increase in Supplemental Nutrition Assistance Program (SNAP) enrollment after the Medicaid expansions. These results are robust to an alternative identification of the most-affected counties, inclusion of counties from comparison states, limiting the control group to only high-poverty counties from comparison states, exclusion of county-specific time trends, and different configuration of clustered errors. My findings provide early insights on the potential effects of new Medicaid expansions of the Affordable Care Act (ACA), since 82 % of those newly eligible are expected to be childless adults.

## Background

Unlike other industrialized nations, the majority of people in the United States are not publicly insured. Public health insurance programs traditionally covered low-income children, their parents, pregnant women, the disabled, and those over age 65. On the other hand, the individual, non-group market used to face significant adverse selection problems before the 2010 Affordable Care Act. Under these circumstances, many Americans could only access affordable private health insurance through their employer. Therefore, expansions of the largest public insurance program, Medicaid, can have a large effect on the labor market, by weakening the link between health insurance and employment.

On July 30th, 1965, Medicaid was enacted as part of the Social Security Amendments, to insure millions of poor individuals. Over the following decades, this program was expanded both at the federal and state levels, becoming the largest public insurance program in the U.S today. The effect of this expansion on labor supply and welfare participation is not easily predictable. Expanded Medicaid coverage may improve health outcomes, thereby increasing labor supply and reducing wages. As individuals work more, they are less likely to participate in other welfare programs, largest of which is the Supplemental Nutrition Assistance Program (SNAP), also known as food stamps. On the other hand, they might have less incentive to work, since they lose eligibility once earned income is over a certain threshold. This in turn would reduce labor supply and increase wages. Welfare participation would also increase as more individuals leave the labor force and become aware of other welfare programs upon enrolling in Medicaid. In this paper, I analyze how Medicaid expansions affect labor force and welfare participation in the U.S.

The next phase of Medicaid expansions already began in 2014 under the Affordable Care Act (ACA), the largest public health insurance expansion since the “Great Society” programs of the 1960s. Those earning less than 138 % of the Federal Poverty Line (FPL) will qualify for Medicaid, regardless of their family or disability status. The June 2012 Supreme Court decision effectively established Medicaid expansions as optional for states, which resulted in 30 states and the District of Columbia opting to expand Medicaid as of September 2015. One way to foresee potential effects of the ACA’s large Medicaid expansions is to analyze the Medicaid expansions of the early 2000s in several states. In August 2001, the Centers for Medicare and Medicaid Services announced the Health Insurance Flexibility and Accountability (HIFA) Demonstration Initiative. HIFA allows states to seek waivers for the various provisions of Medicaid to expand basic health insurance coverage to groups who were not previously eligible, such as childless adults. Arizona, Maine, New Mexico, New York, Oregon, and Vermont used HIFA waivers to expand Medicaid. These states provide an excellent opportunity to analyze the effects of Medicaid expansions.

The contribution of this paper to the literature is threefold. First, this paper includes Medicaid expansions from 6 states representing diverse geographic regions of the country. Second, to my knowledge, this is the first paper to analyze the effects of Medicaid expansions on SNAP participation. Third, this analysis provides insights about the potential effects of the ACA’s Medicaid expansions. Approximately, 82 % of those newly eligible individuals for Medicaid under the ACA are expected to be childless adults. The expansions in the early 2000s insured mostly childless adults, since low-income children along with their parents, and pregnant women were already eligible prior to the expansions.

I use a large data set of 1232 observations from 176 counties over 7 years. Observations start 3 years before the expansion year and end 3 years after the expansion year. To my knowledge, this is one of the largest and longest empirical studies of Medicaid expansions covering 6 states. I combine data from 4 main sources: Local Area Unemployment Statistics, Quarterly Census of Employment and Wages, USDA data on SNAP enrollment, and the U.S. Census. One advantage of a large data set from 6 states is that results are not dependent on individual circumstances of a particular state. Another advantage is that such an analysis is easily replicable for future research since these data sources will be updated every year by the U.S. government as the ACA further expands Medicaid.

My empirical strategy involves a difference-in-differences regression model to estimate the effects of Medicaid expansions on labor supply and welfare participation. My identification strategy is that, if a county has a high poverty rate before the expansion, the county would then be more likely to be affected than counties with low poverty rates since expansions targeted poorer populations. This strategy requires the assumption that in the absence of these expansions, trends in high-poverty and low-poverty counties would not have evolved differently after the expansions. First, I use 4 dependent variables to analyze how expansions affected labor supply and welfare participation: labor force participation rate, average weekly hours, average weekly wages, and enrollment in SNAP. Second, instead of the control group consisting of only low-poverty counties in an expansion state, I also include all counties in a neighboring comparison state that did not expand Medicaid. Finally, I test the robustness of my results by excluding county-specific time trends and clustering standard errors at state- level.

My main result is that individuals have moderately decreased their labor supply after the expansions and increased their participation in SNAP. I find a 1.4 percentage point decrease in labor supply and 0.49 % increase in SNAP enrollment in high-poverty counties after the expansions in comparison to low-poverty counties. Both results are significant at 5 % significance level. Furthermore, I find that average weekly hours decreased by 0.32 h and average weekly wages increased by 1.1 %.

These results are robust to the inclusion of counties from neighboring non-expansion states, limiting the control group to only high-poverty counties from comparison states, exclusion of county-specific time trends, and clustering of standard errors at state-level. Low-income individuals are more likely to quit their job or reduce their work hours to qualify for expanded Medicaid. These individuals are also more likely to sign up for food stamps as they become eligible for Medicaid. My results are moderate in magnitude, smaller than what other papers found by focusing on a single state.

### Related literature

Medicaid expansions of the 1980s and 1990s targeted poor children, their parents, and pregnant women. Therefore, earlier literature on the effects of these expansions on labor supply and welfare participation is limited to these groups. Yelowitz [9] uses the decision by the federal government to separate Medicaid benefits from Aid for Families with Dependent Children (AFDC) program. This separation meant that children and their mothers did not lose Medicaid benefits when they lost AFDC benefits. He finds that Medicaid expansions of the late 1980s led to higher labor supply and lower welfare participation among women with dependent children. Unlike Yelowitz [9], I find lower labor supply and higher welfare participation after the expansions. Medicaid expansions in the 1980s and 1990s allowed children to remain insured even though their mothers earn more income than the threshold for Medicaid eligibility. This type of expansion is likely to create incentives for mothers to work and leave welfare programs. In addition, the expansions in my data set primarily targeted childless adults, whose incentives and behavior would be different than mothers with dependent children.

Gruber and Madrian [7] survey over 50 papers on this topic, and they find, at most, a weak relationship between public health insurance eligibility and labor supply for low-income mothers. By the time of the Medicaid expansions of the early 2000s, low-income mothers, children, and pregnant women were already eligible for Medicaid. Therefore, most of the beneficiaries from these expansions were childless adults. By using data from the early 2000s, I am able to analyze the labor supply and welfare participation of this group. Analysis of this group is quite relevant today, as approximately 82 % of those newly eligible for Medicaid under the ACA are expected to be childless adults. My results indicate a stronger relationship between public health insurance eligibility and labor supply. Benefits of having a job for a mother with dependent children may be higher than those for a childless adult. Therefore, public health insurance eligibility may not translate into changes in labor supply for these mothers to the extent that they would for childless adults.

A recent study, Dave et al. [5] examines the effects of Medicaid expansions in the late 1980s and the early 1990s on the labor supply of pregnant women. Unlike earlier literature, they find a 20 percentage-point increase in Medicaid eligibility was associated with a 6 to 7 % decrease in the probability that a woman who gave birth in the past year was employed. I focus on expansions covering mostly childless adults and find a smaller effect on labor supply. In addition, I expand the analysis by adding welfare participation, rather than focusing only on labor supply. My results find a similar decrease in labor supply, although the magnitude is smaller. Dave et al. [5] analyzed women who gave birth in the past year, while the Medicaid expansions in my data set primarily affected childless adults. For women with children under age 1, Medicaid may be more valuable and that would explain the larger decrease in labor supply.

Another recent paper, Baicker et al. [2] uses Oregon’s lottery to expand Medicaid to randomly selected, low-income, uninsured adults. They find that Medicaid expansions have no effect on employment or earnings, and participation in the food stamp program increases, while other welfare programs are not affected. Similarly, I find that participation in the food stamp program increases, but my results also display a moderate decrease in labor supply with an increase in earnings. A powerful aspect of Baicker et al. [2] is its use of a randomized experiment to expand Medicaid, which is not always available to researchers. I find a similar effect on welfare participation, while finding a negative effect on labor supply. Potentially, there are factors unique to Oregon that affect labor supply in addition to the Medicaid expansions. These factors may not be as prevalent in the 6 states that I study.

Garthwaite et al. [6] exploits the largest Medicaid disenrollment in the history of the United States. In 2005, Tennessee dropped 170,000 childless adults from Medicaid. They find large increases in labor supply, job search behavior, employment, and private insurance coverage in Tennessee following the disenrollment. My results confirm that labor supply is significantly affected by Medicaid eligibility, although I find smaller results. Both Baicker et al. [2] and Garthwaite et al. [6] analyze a single state: Oregon and Tennessee, respectively. Various factors in particular states may affect the results, besides a change in Medicaid eligibility. Therefore, results from a single state may not be generalizable to other states. I include Medicaid expansions from 6 states in my data set: Arizona, Maine, New Mexico, New York, Oregon, and Vermont. These states represent diverse regions of the United States and the results may provide a more comprehensive outlook on the effects of Medicaid expansions.

### Medicaid expansions in the early 2000s

In August 2001, the Centers for Medicare and Medicaid Services (CMS) announced the Health Insurance Flexibility and Accountability (HIFA) Demonstration Initiative. HIFA waivers are variants of traditional Medicaid section 1115 waivers, and they are designed to provide flexibility and federal matching funds to states that want to expand Medicaid to traditionally ineligible groups, primarily childless adults. Atherly et al. [1] reports that 15 states applied and received a HIFA waiver in the early 2000s. However, only 8 of these 15 states implemented large-scale, long-term efforts to reduce the number of uninsured: Arizona, Colorado, Illinois, Michigan, Maine, New Mexico, Oklahoma, and Oregon. Colorado and Oklahoma targeted only pregnant women and the disabled who were previously ineligible. Of the remaining 6 states that expanded Medicaid to childless adults, Atherly et al. [1] found no significant change in insurance coverage in Illinois or Michigan. Both these states had certain restrictions in eligibility that resulted in only a very small share of the population becoming eligible for Medicaid. In addition to the remaining 4 states, New York and Vermont used traditional section 1115 waivers to expand Medicaid to childless adults. Therefore, I use data from Arizona, Maine, New Mexico, New York, Oregon, and Vermont.

Table 1 reports the waiver approval/implementation dates, eligibility requirements, 2007 enrollment, coverage groups other than childless adults, and benefit design of the expansions in 6 expansion states. Arizona and New York were the first states to expand Medicaid to childless adults who earn under 100 % FPL in 2001. In Maine and Vermont, those expansions did not include any groups other than low-income childless adults. Arizona and New York provided full Medicaid benefits, while other states had certain limitations. In New Mexico in 2007, only 7,444 people were enrolled in Medicaid, but Atherly et al. [1] found the third largest increase in insurance coverage in this state, among 8 states in the study, after the Medicaid expansion. Therefore, I include New Mexico as one of my 6 expansion states.

**Table 1:** Medicaid expansions by state

State	Approval	Implementation	Eligibility	HIFA Enrollment 2007	Other groups	Benefits
Arizona	12.12.2001	11.1.2001	<100 % FPL	49, 137	Some parents	Full medicaid
Maine	9.13.2002	10.1.2002	<100 % FPL	17, 449	None	Limited
New Mexico	8.23.2002	7.1.2005	<200 % FPL	7, 444	Some parents	Limited
New York	5.30.2001	10.1.2001	<100 % FPL	98,720	Some parents	Full medicaid
Oregon	10.15.2002	11.1.2002	<100 % FPL	41, 057	Children, pregnant women	Limited
Vermont	9.27.2005	10.1.2005	<150 % FPL	35,700	None	Limited

## Methods

### Data

#### Labor supply and welfare participation

My data set is at county-level and each county-year combination constitutes an observation. I obtained labor supply and welfare participation data for each county in 6 expansion states over 7 years. The first year of observation is three years prior to the year the Medicaid expansion became effective and the last year of observation is three years after the expansion year. Medicaid expansions may take some time to be implemented, since individuals are not immediately aware of their new eligibility. As a robustness check, I also collected the same data for 5 neighboring comparison states that did not expand Medicaid: Nevada as the control state for Arizona, New Hampshire for Maine and Vermont, Colorado for New Mexico, Pennsylvania for New York and Washington for Oregon.

For each county over the 7 years of analysis, I obtained labor force participation rate and average weekly hours worked from the Local Area Unemployment Statistics,^1^ made available by the U.S. Bureau of Labor Statistics. Labor force participation rate is the percentage of the people over age 16 who participate in the labor force, either employed or unemployed. I use these two dependent variables to measure the changes in labor supply after the expansions. In addition, I obtained average weekly wages by county and year from the Quarterly Census of Employment and Wages,^2^ also provided by the U.S. Bureau of Labor Statistics. This dependent variable helps me determine whether the change in wages after the expansions is consistent with the changes in labor supply. I use county-level enrollment in SNAP (food stamps),^3^ provided by the U.S. Department of Agriculture. In fiscal year 2014, this program supplied approximately 46.5 million Americans with an average of $125.35 per person per month in food assistance.

#### Poverty rate and demographics by county

I use the poverty rate by county to identify which counties in an expansion state are most likely to be affected by the expansion, since these expansions mostly covered childless adults under poverty. The U.S. Census reports county poverty rates through Small Area Income and Poverty Estimates (SAIPE).^4^ I regard counties in a state with over 75th percentile of all poverty rates across all counties in that state as my treatment counties. As an alternative identification of treatment counties, I obtained county uninsurance rates from the Small Area Health Insurance Estimates (SAHIE), also published by the U.S. Census Burau.^5^ SAHIE was first launched in 2000, with updated estimates in 2005 and the following years. I use data from 2000 SAHIE, which is a year before the first expansions in Arizona and New York. My results did not change with this alternative identification strategy.

Demographics of a county may have affects on labor supply and welfare participation. The U.S. Census Bureau provides data on county demographics,^6^ where I obtained four county-level demographic variables by year: population, female, black, and Hispanic shares of the population.

#### Summary statistics

Table 2 displays summary statistics of the data analysis. My data set includes 176 counties from 6 states, observed over 7 years: 3 years before the expansion + expansion year + 3 years afterwards. In panel A, I report mean, median, standard deviation and range of all variables, while the medians of the same variables are reported in Panel B for counties by state.

**Table 2:** Summary statistics

Panel A: All counties						
Variable	Obs	Mean	Median	Std. dev.	Min	Max
Number of Counties	176					
Labor force participation rate	1,232	0.58805	0.5928	0.057	0.394	0.784
Average weekly hours worked	1,232	33.3	32.9	2.223	31.2	36.5
Average weekly wages	1,232	536.2465	515.85	115.368	398	748
Food stamps	1,232	5328.50	5,296.50	1,245	2,567	401,684
Poverty rate	1,232	12.3215	11.875	3.8855	3.8	38.9
Female	1,232	50.9	51.2	1.2	48.3	53.5
Black	1,232	3.097	0.95	5.7	0	43
Hispanic	1,232	7.1725	2.945	10.8775	0.4	81.9
Panel B: Counties by state						
State	AZ	ME	NM	NY	OR	VT
Number of Counties	15	16	33	62	36	14
Labor Force Participation Rate	0.534	0.629	0.618	0.621	0.624	0.689
Average weekly hours worked	33.6	31.8	32.1	34.1	32.4	32
Average weekly wages	501	518	505	556	527.5	582
Food stamps	12*313	6,420.5	4,979.2	5,235	4,094.5	3,368.5
Poverty rate	15.6	10.1	18	13.1	13.2	10.7
Female	50.1	51.2	50.3	50.7	50.3	50.4
Black	1.1	0.4	0.4	3.8	0.4	0.5
Hispanic	25.9	0.7	42.1	15.1	8	0.9

*Each county is observed for 7 years, leading to 1,232 observations

Mean labor force participation rate was around 59 %, with the lowest in Arizona and the highest in Vermont. Average weekly hours were 33.3 h, with the lowest in Maine and the highest in New York. Average weekly wage was around $536, with the lowest in Arizona and the highest in Vermont. Approximately 5,328 people were on food stamps in an average county, with the highest participation in Arizona and the lowest in Vermont. New Mexico and Arizona had higher poverty rates than the other states. New York had the highest share of African Americans per county, while New Mexico and Arizona had the highest share of Hispanics.

**Fig. 1:**
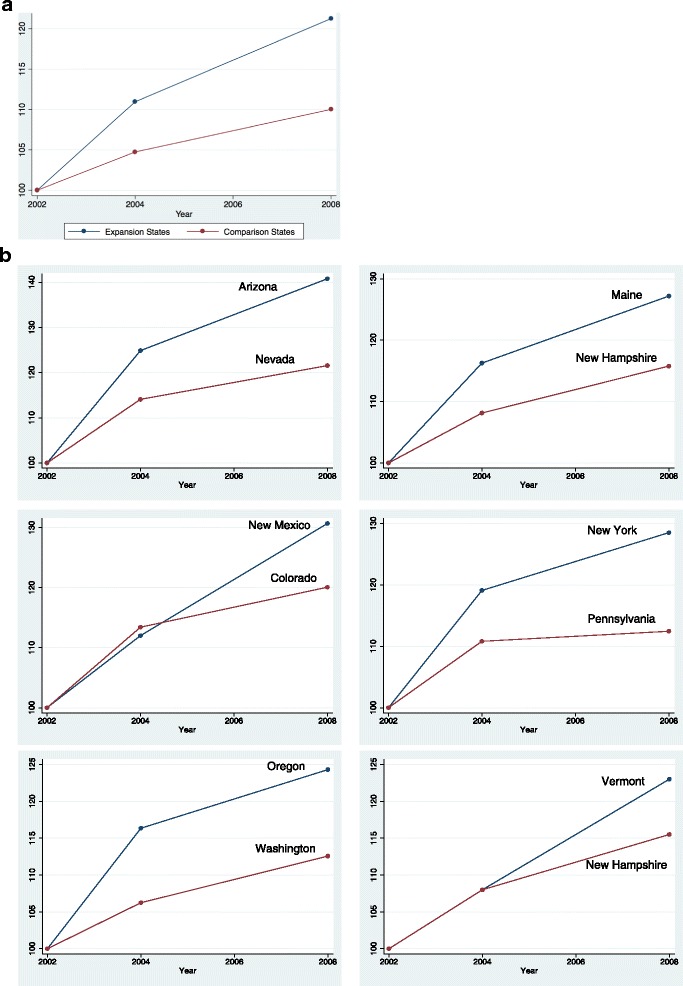
**a**. Total Medicaid enrollment in expansion and comparison states as a percentage of 2002 enrollment. Source: The Medicaid Analytical Extract (MAX) data, produced by the Centers for Medicare & Medicaid Services. **b**. Medicaid enrollment by each expansion state and its comparison state as a percentage of 2002 enrollments. Source: The Medicaid Analytical Extract (MAX) data, produced by the Centers for Medicare & Medicaid Services

Figures 1a and 1b display Medicaid enrollment as a percentage of 2002 enrollment in both expansion and comparison states. Enrollment has been steadily increasing for both groups, but expansion states report more than double the increase in comparison states during the early 2000s. Total Medicaid enrollment in the 6 expansion states increased by about 23 versus 10 % in comparison states. Such a change in Medicaid enrollment may have effects on labor supply and welfare participation.

**Fig. 2:**
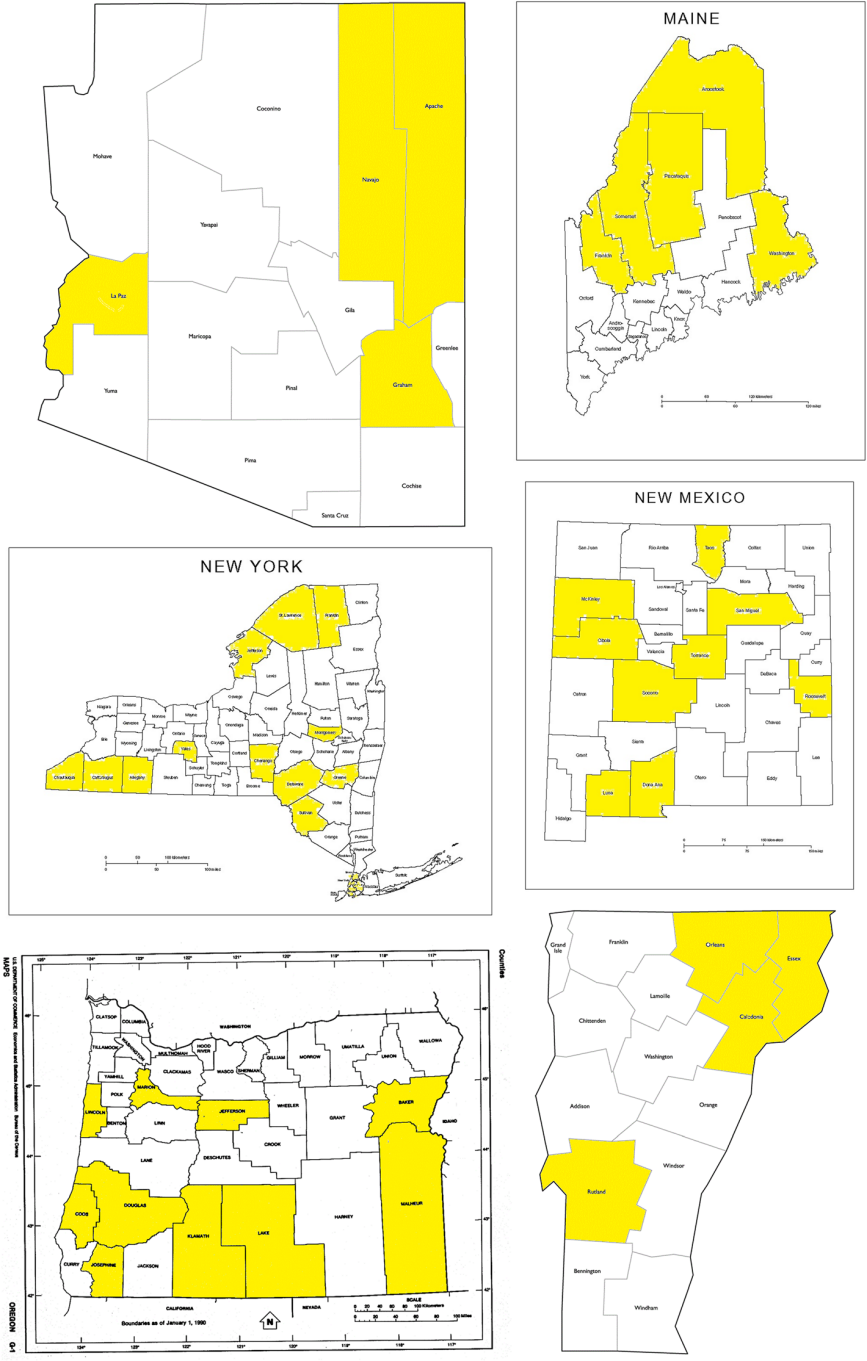
County maps by poverty rate

Figure 2 includes a map of the 6 states in my data set. Shaded counties have higher than 75th percentile of poverty rates across all counties in the same state. They constitute my treatment group. Other counties are my control group, as Medicaid expansions are less likely to affect those counties.

### Econometric Method

This section presents my main empirical results. I first introduce the difference-in-differences model in section Economic method. I then examine changes in labor supply (section Labor supply). In sections Average weekly hours and Average weekly wages, I demonstrate how Medicaid expansions affected average weekly hours and average weekly wages, respectively. In section SNAP enrollment, I estimate the effects of these expansions on SNAP enrollment. Finally, in section Robustness, I analyze the robustness of my results to the inclusion of counties from neighboring non-expansion states, exclusion of county-specific time trends, and clustering of standard errors at state-level.

First, I estimate the following difference-in-differences regression model to measure the effects of Medicaid expansions on 4 dependent variables:

1 $$ \begin{array}{l}{y}_{ct}={\alpha}_c+{\delta}_t+{\beta}_1Pos{t}_tx\; Povert{y}_{ct}+{\beta}_2Pos{t}_t+{\beta}_3 Povert{y}_{ct}+{\beta}_5{X}_{ct}+{\beta}_6 Implemen{t}_t+\hfill \\ {}{\beta}_7 Implemen{t}_t\;x\kern0.24em  Povert{y}_{ct}+{\displaystyle \sum_{c=1}^{176}{\beta}_{8c}x\; Count{y}_c}\;x\;t+{\varepsilon}_{ct}\hfill \end{array} $$

where the variable*y*_*ct*_represents one of the 4 outcomes for county c and year t: labor force participation rate, average weekly hours, average weekly wages, and SNAP enrollment. is a binary variable with value 1 after the Medicaid expansions are implemented; *Poverty*_*ct*_is the poverty rate of county c in year t; is a set of variables representing county demographics (population, gender, poverty, and race); is a binary variable with value 1 during the implementation years; and is the county-specific time trend. The model includes county fixed effects (*α*_*c*_), year fixed effects (*δ*_*t*_), and an error term (*ε*_*ct*_)that is assumed to be uncorrelated with other unobserved determinants of the outcome variable.

The main coefficient of interest is *β*_1_, which is the difference-in-differences estimate of the effect of the Medicaid expansion. A negative and statistically significant coefficient would indicate that counties with higher poverty rates had a decrease in labor force participation (or other dependent variables) after the Medicaid expansions, as more people quit their jobs to qualify for expanded Medicaid.

I use two different specifications of (*Post*_*t*_) to identify the post-expansion period: *Post1* includes the second and third year after the expansion year, while *Post2* includes only the third year. There are two reasons for these alternative specifications. First, states implemented Medicaid expansions in varying months and years. For example, Arizona’s Medicaid expansion was implemented in November 2001, while Vermont’s expansion became effective in October 2005. It can take more than a year for a significant portion of the newly eligible population to participate in this expansion, since many newly eligible, uninsured, childless adults do not become immediately aware of these expansions. Second, it can also take considerable time for the expansions to affect labor supply and welfare participation, since some individuals might delay making such major decisions to become eligible for Medicaid. In accordance with the post-expansion period, I use two alternative specifications to identify the implementation period: *Implement*1_*t*_and *Implement*2_*t*_. The former takes the value 1 for both in the year Medicaid expansion became effective along with the following year, while the latter also includes the 2 years after the expansion.

One concern with county-level analyses is that recessions or contemporaneous policy changes at both federal and state levels can differentially affect some counties. One advantage of this data set is that it includes data from the late 1990s (AZ and NY) to early 2008 (VT). This time period provides three full years of data after a state’s Medicaid expansion, but avoids potential confounding effects arising from the 2008 recession, which started to affect the labor market in the second half of 2008 [3].

In an alternative specification of eq. (1), I replace *Poverty*_*ct*_ with *Treated*_*ct*_, which is a binary variable indicating that the observed county was one of the poorest counties in the expansion state (over 75th percentile of all county poverty rates in the same state). This alternative specification is my preferred specification for two reasons. First, the continuous variable, *Poverty*_*ct*_, assumes a linear relationship between county poverty rate and the dependent variable. Second, the results would be more robust to measurement errors in *Poverty*_*ct*_, if a binary variable is used.

## Results

### Labor supply

**Fig. 3:**
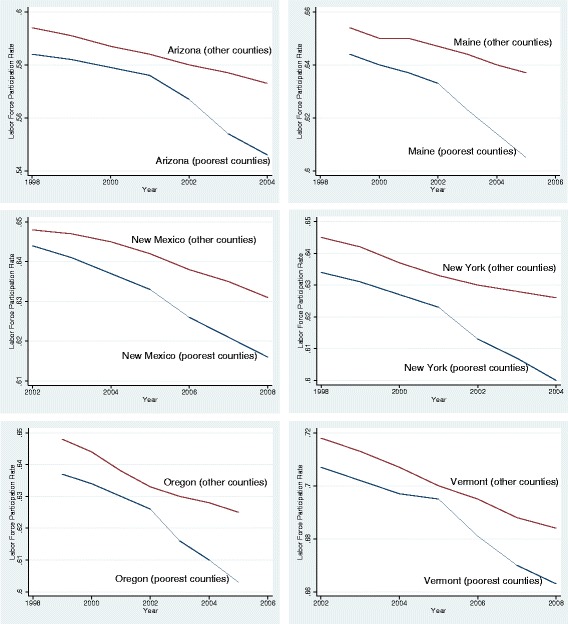
Pre and post-expansion trends for labor force participation rate

I start with examining the trends in labor force participation rate before and after the expansions. Figure 3 presents the share of residents participating in the labor force for each state. In all expansion states, labor force participation rate was evolving similarly before the expansions in both poorest counties and other counties. After the expansions, however, I observe a faster decline in labor force participation rate in the poorest counties of 5 states, with the share of residents in the labor force of the poorest counties dropping by roughly 1 percentage point more than those in other counties. By contrast, trends in other counties were similar to pre-expansion trends.

Table 3 displays the estimates of eq. (1), where the dependent variable is the labor force participation rate. In all four specifications, I control for county fixed effects, year fixed effects, county demographics, and county-specific time trend. Columns 1 and 2 show the results for the continuous version of county poverty, for the first and second versions of *Post*_*t*_/*Implement*_*t*_, respectively. Main variables of interest are the interactions between *Poverty*_*ct*_ and the two versions of *Post*_*t*_: Post1*Poverty and Post2*Poverty. In general, coefficients are small, negative, and statistically significant. Column 1 indicates that a county with 1 percentage-point higher poverty rate than another county in the same state would have 0.13 percentage point drop in labor force participation rate two and three years after the expansion. This effect is statistically significant at 5 % significance level. When the post-expansion period is defined as only the third year after the expansion year, Column 2 indicates that the drop in labor force participation rate is slightly higher: 0.16 percentage points and also statistically significant at 5 % significance level. This translates into a 1.6 percentage point drop in labor force participation for a county with a 10 percentage-point higher poverty rate. I do not observe any significant effects on labor force participation at 5 % significance level during the implementation years.

**Table 3:** Estimated effects of medicaid expansions on labor force participation rate dependent variable: Labor force participation rate

Independent variable	(1)	(2)	(3)	(4)
Post1	−0.011	–	−0.009	–
	(0.012)	–	(0.010)	–
Implement1	−0.015	–	−0.013	–
	(0.069)	–	(0.064)	–
Post1*Poverty	−0.13**	–	–	–
	(0.06)	–	–	–
Implement1*Poverty	−0.09*	–	–	–
	(0.05)	–	–	–
Post2	–	−0.015	–	−0.012
	–	(0.013)	–	(0.011)
Implement2	–	−0.014	–	−0.014
	–	(0.012)	–	(0.090)
Post2*Poverty	–	−0.16**	–	–
	–	(0.07)	–	–
Implement2*Poverty	–	−0.08*	–	–
	–	(0.05)	–	–
Post1*Treated	–	–	−1.5**	–
	–	–	(0.60)	–
Implement1*Treated	–	–	_−_0.9**	–
	–	–	(0.40)	–
Post2*Treated	–	–	–	−1.4***
	–	–	–	(0.50)
Implement2*Treated	–	–	–	−0.8*
	–	–	–	(0.05)
Constant	0.3116	0.285	0.3233	0.2978
	(0.2867)	(0.3456)	(0.3187)	(0.3320)
County demographics	Yes	Yes	Yes	Yes
County fixed-effects	Yes	Yes	Yes	Yes
County-specific time trend	Yes	Yes	Yes	Yes
N	1232	1232	1232	1232
R^2^	0.74	0.75	0.71	0.72

Standard errors are clustered by county and given in parantheses. *indicates *p* < 0.10, **indicates *p* < 0.05, ***indicates *P* < 0.01

Columns 3 and 4 include the binary version of county poverty (*Treated*_*c*_), for the first and second versions of*Post*_*t*_/*Implement*_*t*_, respectively. Main variables of interest are the interactions between *Treated*_*c*_ and the two versions of*Post*_*t*_: Post1*Treated and Post2*Treated. As in the first two columns, coefficients are small, negative, and statistically significant. Column 3 displays that a high-poverty county had 1.5 percentage point lower labor force participation rate two and three years after the expansion. This effect is statistically significant at 5 % significance level. Column 4 reports a 1.4 percentage point drop when the post-expansion period includes only the third year after the expansion year. This effect is statistically significant even at 1 % significance level.

### Average weekly hours

**Fig. 4:**
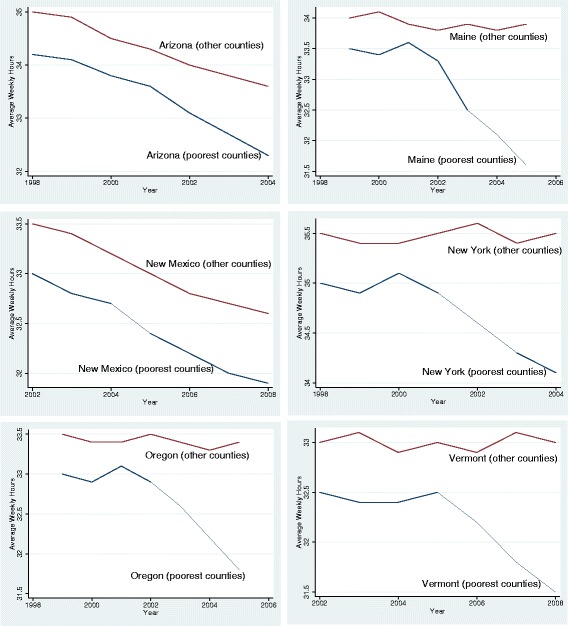
Pre and post-expansion trends for average weekly hours worked

In order to qualify for expanded Medicaid, some individuals may choose to reduce their weekly hours rather than dropping out of the labor force. By doing so, their earnings would still be below the maximum income thresholds for Medicaid. Such a choice by a part of the population can translate into reduced average weekly hours in that county. In Fig. 4, I plot the trends in average weekly hours between poorest counties and other counties over time. Before the expansions, average weekly hours were stable over time in both sets of counties in 4 of the 6 expansion states. In New Mexico and Arizona, however, there was a slight downward trend in average weekly prior to the expansions. Following the expansions, I observe a further decline in average weekly hours in every state except New Mexico. In New Mexico, the same trends before the expansions continue after the expansions. On average, I find a 0.2 h decline per week in average weekly hours. Since the average weekly hours per county was 33.3 h in my data set, this corresponds to a 0.6 % decrease in average weekly hours after the expansions.

Table 4 reports the estimates of eq. (1), where the dependent variable is the average weekly hours. Similar to Table 3, I control for county fixed effects, year fixed effects, county demographics, and county-specific time trend. Columns 1 and 2 show the results for the continuous version of county poverty, for the first and second versions of *Post*_*t*_/*implement*_*t*_, respectively. In general, coefficients are small, negative, and statistically significant. Column 1 indicates that a county with 1 percentage-point higher poverty rate than other counties in the same state would have 0.033 h per week decrease in average weekly hours two and three years after the expansion. This effect is statistically significant at 5 % significance level. With the second version of the post-expansion period, Column 2 indicates that the drop in average weekly hours is slightly lower: 0.031 h and also statistically significant at 5 % significance level. This translates into a 0.9 % drop in labor force participation for a county with 10 percentage point higher poverty rate. I also observe significant, yet small effects on average weekly hours at 5 % significance level during the implementation years.

**Table 4:** Estimated effects of medicaid expansions on average weekly hours dependent variable: Average weekly hours

Independent variable	(1)	(2)	(3)	(4)
Post1	0.023	–	0.019	–
	(0.014)	–	(0.015)	–
Implement1	0.015	–	0.012	–
	(0.048)	–	(0.044)	–
Post1*Poverty	−0.033**	–	–	–
	(0.014)	–	–	–
Implement1*Poverty	−0.025*	–	–	–
	(0.015)	–	–	–
Post2	–	0.017	–	0.25
	–	(0.012)	–	(0.017)
Implement2	–	0.013	–	0.16
	–	(0.042)	–	(0.044)
Post2*Poverty	–	−0.031***	–	–
	–	(0.012)	–	–
Implement2*Poverty	–	−0.022*	–	–
	–	(0.013)	–	–
Post1*Treated	–	–	−0.29**	–
	–	–	(0.014)	–
Implement1*Treated	–	–	−0.23**	–
	–	–	(0.011)	–
Post2*Treated	–	–	–	−0.32**
	–	–	–	(0.013)
Implement2*Treated	–	–	–	−0.26**
	–	–	–	(0.015)
Constant	0.2875	0.2789	0.2684	0.2569
	(0.2267)	(0.2352)	(0.2411)	(0.2173)
County demographics	Yes	Yes	Yes	Yes
County fixed-effects	Yes	Yes	Yes	Yes
County-specific time trend	Yes	Yes	Yes	Yes
N	1232	1232	1232	1232
_R_2	0.53	0.54	0.57	0.59

Standard errors are clustered by county and given in parantheses. *indicates *p* < 0.10, **indicates *p* < 0.05, ***indicates *P* < 0.01

Columns 3 and 4 include the binary version of county poverty (*Treated*_*c*_), for the first and second versions of *Post*_*t*_/*Implement*_*t*_, respectively. Similar to the first two columns, coefficients are small, negative, and statistically significant. Column 3 displays high-poverty counties have 0.29 h lower average weekly hours, two and three years after the expansion. This effect is statistically significant at 5 % significance level. Column 4 reports a 0.32 h drop when the post-expansion period includes only the third year after the expansion year. This effect is statistically significant even at 1 % significance level.

### Average weekly wages

**Fig. 5:**
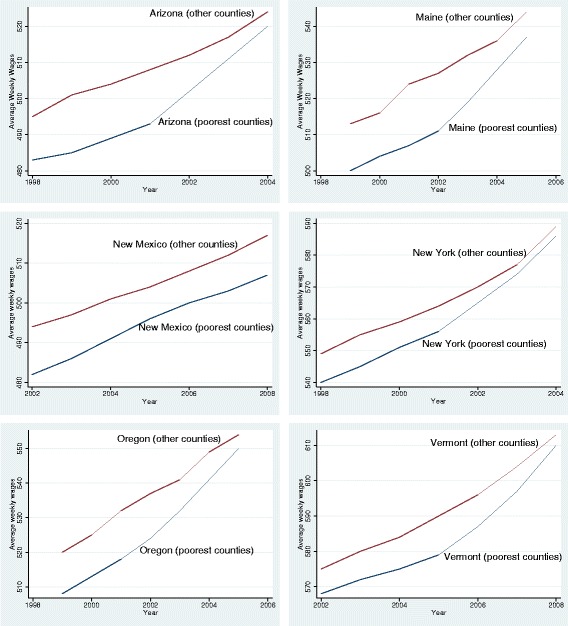
Pre and post-expansion trends for average weekly wages

Since Medicaid expansions changed the eligibility criteria for individuals, rather than changing incentives of employers, I expect the expansions to affect labor supply rather than labor demand. I evaluate this by analyzing the changes in average weekly wages, since a decrease in labor supply would suggest an increase in wages for the remaining employees. Figure 5 plots the average weekly wages over time for the poorest counties and other counties. In 5 of the 6 states, I observe a slight increase in wages after the expansions in poorest counties. Before the expansions, average weekly wages evolve similarly. I observe around 1 % increase in average weekly wages after the expansions.

Table 5 displays the estimated effects of Medicaid expansions on natural logarithm of average weekly wages. Coefficients on the interactions of post-expansion period and county poverty are small, positive, and statistically significant at 5 % significance level. In column 1, I observe an increase in average weekly wages of 0.9 %, two and three years after the expansions, when a county has 10-percentage point more poverty. For the second version of post-expansion period, the same effect is 0.8 % in Column 2. Since the average weekly wages in my data set is $536, this would suggest a $4.3 increase in wages in the poorest counties.

**Table 5:** Estimated effects of medicaid expansions on average weekly wages dependent variable: Log (average weekly wages)

Independent variable	(1)	(2)	(3)	(4)
Post1	0.0017	–	0.014	–
	(0.013)	–	(0.015)	–
lmplementl	0.0014	–	0.016	–
	(0.046)	–	(0.043)	–
Post1*Poverty	0.0009**	–	–	–
	(0.004)	–	–	–
Implement1*Poverty	0.0007*	–	–	–
	(0.004)	–	–	–
Post2	–	0.0015	–	0.016
	–	(0.012)	–	(0.012)
lmplement2	–	0.0013	–	0.015
	–	(0.039)	–	(0.043)
Post2*Poverty	–	0.0008**	–	–
	–	(0.004)	–	–
lmplement2*Poverty	–	0.0006*	–	–
	–	(0.005)	–	–
Post1*Treated	–	–	0.012**	–
	–	–	(0.006)	–
Implement1*Treated	–	–	0.006**	–
	–	–	(0.003)	–
Post2*Treated	–	–	–	0.011**
	–	–	–	(0.005)
Implement2*Treated	–	–	–	0.008*
	–	–	–	(0.005)
Constant	0.2631	0.2678	0.2613	0.2436
	(0.2371)	(0.2392)	(0.2387)	(0.2233)
County demographics	Yes	Yes	Yes	Yes
County fixed-effects	Yes	Yes	Yes	Yes
County-specific time trend	Yes	Yes	Yes	Yes
N	1232	1232	1232	1232
R^2^	0.57	0.59	0.63	0.64

Standard errors are clustered by county and given in parantheses. *indicates *p* < 0.10, **indicates *p* < 0.05, ***indicates *P* < 0.01

I observe similar small but positive effects when I use a binary version of county poverty. Average weekly wages increase by 1.2 % in poorest counties two and three years after the expansions (Column 3). This is also significant at 5 % significance level. When I restrict the post-expansion period to only the third year after the expansions, Colum 4 indicates that the same effect is 1.1 %. Therefore, I find evidence of a slight wage increase following the expansions. The decrease in labor supply as some individuals drop out of the labor force to qualify for expanded Medicaid translates into higher wages for the remaining employees in poorest counties. Another factor that may be in play here is that companies have an incentive to pay their employees more when they do not have to finance insurance of some employees, since they now obtain insurance from expanded Medicaid.

### SNAP enrollment

Since Medicaid is a means-tested program, participation in this program may increase the awareness of other means-tested programs. The largest welfare program, by number of participants in the US is the Food Stamp Program (SNAP). In fiscal year 2014, roughly 46.5 million Americans received an average of $125.35 per month in food assistance.^7^ Even though this program has many beneficiaries, literature on the effects of Medicaid expansions on SNAP is very limited.

**Fig. 6:**
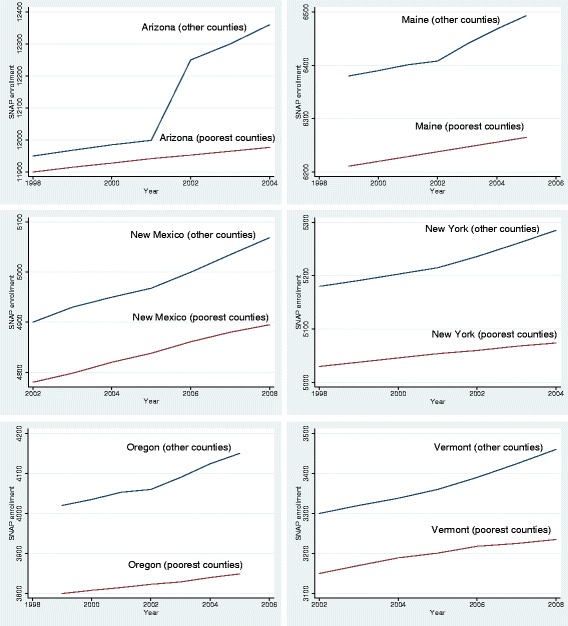
Pre and post-expansion trends for Supplemental Nutritional Assistance Program Enrollment

In Fig. 6, I observe an initial increase in food stamps participation followed by stabilization. There was an 8-month long recession in 2001, which explains the initial increase. After the recession of 2001, GDP growth rates remained positive each year until 2008. The poorest counties in all states display a small increase in SNAP enrollment after the expansions.

Table 6 reports the regression estimates with natural logarithm of SNAP enrollment as the dependent variable. Interaction of the post-expansion period and county poverty has small, positive, and statistically significant coefficients at 5 % significance level. Column 1 indicates SNAP enrollment increases by 0.6 %, two and three years after the expansions, when a county has 10-percentage points more poverty than other counties. For the second version of the post-expansion period, I observe a 0.5 % increase in Column 2.

**Table 6:** Estimated effects of medicaid expansions on food stamps dependent variable: Lou (food stamp enrollment)

Independent variable	(1)	(2)	(3)	(4)
Post1	0.015	–	0.014	–
	(0.012)	–	(0.013)	–
Implement1	0.011	–	0.010	–
	(0.038)	–	(0.031)	–
Post1*Poverty	0.0006**	–	–	–
	(0.0003)	–	–	–
Implement1*Poverty	0.0008*	–	–	–
	(0.0005)	–	–	–
Post2	–	0.013	–	0.013
	–	(0.011)	–	(0.013)
lmplement2	–	0.012	–	0.015
	–	(0.035)	–	(0.041)
Post2*Poverty	–	0.0005**	–	–
	–	(0.0002)	–	–
lmplement2*Poverty	–	0.0007*	–	–
	–	(0.0004)	–	–
Post1*Treated	–	–	0.0052**	–
	–	–	(0.0024)	–
Implement1*Treated	–	–	0.007**	–
	–	–	(0.0030)	–
Post2*Treated	–	–	–	0.0049**
	–	–	–	(0.0023)
lmplement2*Treated	–	–	–	0.0042*
	–	–	–	(0.0026)
Constant	0.2512	0.2567	0.2641	0.2444
	(0.2163)	(0.2281)	(0.2114)	(0.2567)
County demographics	Yes	Yes	Yes	Yes
County fixed-effects	Yes	Yes	Yes	Yes
County-specific time trend	Yes	Yes	Yes	Yes
N	1232	1232	1232	1232
R^2^	0.48	0.49	0.51	0.52

Standard errors are clustered by county and given in parantheses. *indicates *p* < 0.l0. ** indicates *p* < 0.05. ***indicates *P* < 0.01

Binary version of county poverty also yields small but positive effects. SNAP enrollment increases by 0.52 % two and three years after the expansions (Column 3), and this is significant at 5 % significance level. With the second version of poverty, Column 4 reports a 0.49 % increase. In all regression specifications, I find increases in SNAP enrollment following the expansions. As individuals leave the labor force, sign up for Medicaid, and become more aware of other welfare programs, they are more likely to enroll in SNAP.

### Robustness

My identification strategy involves comparing counties by poverty rate, as counties with the highest poverty rates are likely to have a greater number of individuals who become eligible for Medicaid after the expansions. Another way to expand the control group of counties is to include counties from a neighboring comparison state that did not expand Medicaid. This, along with counties in the expansion state that are less than 75th percentile of the poverty rate, will make up the control group. In this case, treated counties are the counties in the expansion state that have greater than 75th percentile of the poverty rate across all counties in that state. I report the results with this expanded control group of counties in the first column of Table 7. Each row corresponds to a separate regression and I display only the coefficient for the main variable of interest. My results are robust to the inclusion of counties from comparison states as part of my control group. Labor force participation and average weekly hours show small but significant decreases after the Medicaid expansions. In addition, average weekly wages and SNAP enrollment show an increase.

**Table 7:** Robustness of the results

Dependent variable	Neighboring states	High-poverly counties	No time trend	Clustering at state-level
Labor force participation rate - Post2*Treated	−1.16**	−1.19***	−1.24**	−1.23**
	(0.54)	(0.45)	(0.58)	(0.61)
Average weekly hours - Post2*Treated	−0.29**	−0.24**	−0.31**	−0.28**
	(0.014)	(0.012)	(0.015)	(0.013)
Log (weekly wages)-Post2*Treated	0.012**	0.013**	0.014*	0.010**
	(0.006)	(0.007)	(0.008)	(0.005)
Log (food stamp enrollment)-Post2*Treated	0.0045**	0.0042**	0.0047**	0.0043**
	(0.0022)	(0.0020)	(0.0023)	(0.0021)
County demographics	Yes	Yes	Yes	Yes
County fixed-effects	Yes	Yes	Yes	Yes
County-specific time trend	Yes	Yes	No	Yes
N	2604	651	1232	1232
R^2^	0.45	0.47	0.44	0.46

Standard errors are clustered by county except for last column and given in parantheses. *indicates *p* < 0.10, **indicates *p* < 0.0 ***indicates *P* < 0.01

There may be different trends in high-poverty counties versus low-poverty counties. If these trends were driving the results, then defining the control group as a mix of low-poverty counties in an expansion state and high-poverty counties in a comparison state would confound the results. To address this potential problem, I restrict my control group to only the high-poverty counties in a comparison state. With this restriction, I compare the effects of the Medicaid expansions on high-poverty counties in relation to the trends in high-poverty counties in neighboring states that did not expand Medicaid during the same period. I report the results with this restricted control group in the second column of Table 7. Results are very similar and I do not have evidence to claim that the choice of control group is driving the results.

Difference-in-differences approach does not require county-specific time trends to be included as control variables. One way to check the robustness of my results is to rerun the regressions without the county-specific time trends. The third column of Table 7 includes the coefficients for my main variable of interest without any county-specific time trends. For labor force participation rate, average weekly hours, and SNAP enrollment, the coefficients still have the same sign and significance. On the other hand, although average weekly wages still increase following the Medicaid expansions, this effect is only statistically significant at 10 % significance level.

If an omitted variable causes correlation between observations across counties, then the error terms would also demonstrate this correlation. I address this potential issue by clustering standard errors at county-level in all my regressions. However, there are large differences in the underlying population in each county and this clustering approach may not be valid [8]. One way to check robustness of my results is to cluster standard errors by state, although clustering on only a few states is not always optimal [4]. I report the results with standard errors clustered at state-level in the last column of Table 7 where all the coefficients still have the same sign and significance level. Therefore, I rule out the possibility that the precision of standard errors is driving the results.

## Discussion

### Potential for selection bias

Prior to the Medicaid expansions, the poorest counties in expansions states may have already had different characteristics in labor supply and welfare participation. If this is the case, then the regression results may reflect selection bias. I compare the poorest counties in expansion states to the poorest counties in comparison states for the mean value of 4 dependent variables. The difference between two means for each of the 4 dependent variables is statistically insignificant at 5 % significance level and I fail to reject the hypothesis that poorest counties in the expansion states are different than the poorest counties in comparison states.

### Potential for omitted variable bias

Ideally, I would prefer to control for other economic factors that can affect the 4 dependent variables. However, such economic factors are likely to be determined endogenously with labor force participation, average weekly hours, wages, and SNAP enrollment. Inclusion of a control variable, such as unemployment rate, may bias the results due to this endogeneity. One way to mitigate this omitted variable concern is to include both county and year fixed effects, which I do in all specifications of my regressions. These fixed effects would account for the factors that are unique within a county and across counties within the same year.

## Conclusions

This research shows that Medicaid expansions in 6 states led to small, but significant effects on labor force participation, average weekly hours, wages, and SNAP enrollment. After controlling for county fixed effects, year fixed effects, county-specific time trend, and four county-level demographic variables, I find 1.4 % decrease in labor force participation rate, 0.32 h decrease in working hours, 1.1 % increase in wages, and 0.49 % increase in SNAP enrollment in poorest counties of 6 expansion states. In addition, I expand my control counties to include all counties in a neighboring comparison state. Results are robust to the inclusion of these counties in comparison states, limiting the control group to only high-poverty counties from comparison states, exclusion of county-specific time trends, and clustering standard errors at state-level.

One limitation of this research is that Medicaid expansions affected a small share of the population in each county and data is not available at individual-level for the 4 dependent variables. Individual-level data do not disclose each individual’s county due to privacy concerns. Therefore, I am unable to analyze labor supply and welfare participation of individuals who actually gained Medicaid after these expansions. However, I still find small, but significant effects on labor supply and welfare participation at county-level. Even though the expansions affect a small share of the population, I observe county-level effects due to changes in labor supply and welfare participation of the expansion population.

My findings provide support for Medicaid expansions’ moderate effects on labor supply and welfare participation, as individuals become more likely to leave the labor force and enroll in welfare programs. This result implies that expanding Medicaid to childless adults under the Affordable Care Act may lead to a slight decrease in labor force participation and an increase in enrollment of other welfare programs. However, as millions of previously uninsured people gain insurance under the ACA, benefits of having insurance may outweigh the negative and moderate effects on labor supply and welfare participation. Since the major provisions of the ACA have been implemented in 2014, it would take a few more years to observe how these factors play out in the labor market.

## Footnotes

1 Data and documentation are available from the BLS website: http://www.bls.gov/lau/

2 Data and documentation are available from the BLS website: http://www.bls.gov/cew/

3 Data and documentation are available from the USDA website: http://www.fns.usda.gov/pd/supplemental-nutrition-assistance-program-snap

4 Data and documentation are available from the US Census Bureau website: http://www.census.gov/did/www/saipe/

5 Data and documentation are available from the US Census Bureau website: http://www.census.gov/did/www/sahie/

6 Data and documentation are available from the US Census Bureau website: https://www.census.gov/support/USACdataDownloads.html

7 "SNAP Monthly Data"*. Fns.usda.gov. 6 February 2015*.

## References

[1] Atherly A, Dowd BE, Coulam RF, Guy G (2012). The Effect of HIFA Waiver Expansions on Uninsurance Rates in Adult Populations. Health Serv Res.

[2] Baicker K, Taubman S, Allen H, Bernstein M, Gruber J, Newhouse J, Schneider E, Wright B, Zaslavsky A, Finkelstein A. The Oregon Experiment – Effects of Medicaid on Clinical Outcomes. N Engl J Med. 2013;368:1713–22. 10.1056/NEJMsa1212321 PMC3701298 23635051

[3] Business Cycle Dating Committee Report. National Bureau of Economic Research. 2010. Available at http://www.nber.org/cycles/sept2010.html.

[4] Cameron AC, Gelbach JB, Miller DL (2008). Bootstrap-based improvements for inference with clustered errors. Rev Econ Stat.

[5] Dave DM, Decker S, Kaestner R, Simon K (2015). The Effect of Medicaid Expansions in the late 1980s and early 1990s on the Labor Supply of Pregnant Women. Am J Health Econ.

[6] Garthwaite C, Gross T, Notowidigdo MJ (2014). Public Health Insurance, Labor Supply, and Employment Lock. Q J Econ.

[7] Gruber J, Madrian BC, McLaughlin C (2004). Health Insurance, Labor Supply, and Job Mobility: A Critical Review of the Literature. Health Policy and the Uninsured.

[8] MacKinnon, J. G. & Webb, M. D. (2015). “Wild bootstrap inference for wildly different cluster sizes", QED Working Paper No. 1314.

[9] Yelowitz AS (1995). The Medicaid Notch, Labor Supply and Welfare Participation: Evidence from Eligibility Expansions. Q J Econ.

